# Morphological and physiological retinal degeneration induced by intravenous delivery of vitamin A dimers in rabbits

**DOI:** 10.1242/dmm.017194

**Published:** 2014-12-12

**Authors:** Jackie Penn, Doina M. Mihai, Ilyas Washington

**Affiliations:** Columbia University Medical Center, Ophthalmology, New York, NY 10032, USA.

**Keywords:** Vitamin A, Neurodegeneration, Bisretinoids, A2E, RPE, Vitamin A dimer, Age-related macular degeneration, AMD, Stargardt

## Abstract

The eye uses vitamin A as a cofactor to sense light and, during this process, some vitamin A molecules dimerize, forming vitamin A dimers. A striking chemical signature of retinas undergoing degeneration in major eye diseases such as age-related macular degeneration (AMD) and Stargardt disease is the accumulation of these dimers in the retinal pigment epithelium (RPE) and Bruch’s membrane (BM). However, it is not known whether dimers of vitamin A are secondary symptoms or primary insults that drive degeneration. Here, we present a chromatography-free method to prepare gram quantities of the vitamin A dimer, A2E, and show that intravenous administration of A2E to the rabbit results in retinal degeneration. A2E-damaged photoreceptors and RPE cells triggered inflammation, induced remolding of the choroidal vasculature and triggered a decline in the retina’s response to light. Data suggest that vitamin A dimers are not bystanders, but can be primary drivers of retinal degeneration. Thus, preventing dimer formation could be a preemptive strategy to address serious forms of blindness.

## INTRODUCTION

Age-related macular degeneration (AMD) is the leading cause of unpreventable blindness. With increasing global populations and falling death rates, the prevalence of AMD is predicted to rise. Although chemical signatures of the aging and degenerating retina have been described, a pressing challenge is determining which signatures are primary drivers of the degenerative process and which are secondary symptoms. The main drivers responsible for the genesis of AMD have been elusive, resulting in approaches that intervene presumably late in disease pathogenesis, by, *inter alia*, targeting angiogenesis (Bressler, 2009), inflammation (Augustin and Kirchhof, 2009; Kanda et al., 2008), apoptosis (Dunaief et al., 2002) or cholesterol/lipid metabolism (Curcio et al., 2009; Curcio et al., 2011; Pikuleva and Curcio, 2014; Spaide et al., 2003).

To sense light, the retina utilizes vitamin A in a process called the visual cycle. During this cycle, a fraction of vitamin A dimerizes. One of the most striking and consistent chemical signature of the aging retina and retinas of people affected with juvenile retinal degenerations such as Stargardt disease, is the accumulation of these dimers in the retinal pigment epithelium (RPE) (Eldred and Katz, 1988; Eldred and Lasky, 1993; Mihai and Washington, 2014; Sakai et al., 1996) and underlying Bruch’s membrane (BM) (Murdaugh et al., 2010). Dimerized vitamin A has been proposed as a primary trigger of retinal degeneration (Eldred, 1995). If true, methods to prevent either vitamin A dimerization or the accumulation of these dimers might provide therapeutics that intervene early in disease pathogenesis to prevent vision loss. Although biochemical mechanisms by which vitamin A dimers, such as A2E, might induce retinal toxicity have been proposed (Anderson et al., 2013; Avalle et al., 2004; Ben-Shabat et al., 2002a; Ben-Shabat et al., 2002b; Bergmann et al., 2001; De and Sakmar, 2002; Dontsov et al., 2009; Finnemann et al., 2002; Holz et al., 1999; Iriyama et al., 2008; Iriyama et al., 2009; Kanofsky et al., 2003; Lakkaraju et al., 2007; Lukiw et al., 2006; Ma et al., 2013; Moiseyev et al., 2010; Murdaugh et al., 2009; Murdaugh et al., 2010; Nowak, 2013; Radu et al., 2004; Roberts et al., 2002; Sokolov et al., 2007; Sparrow, 2010; Sparrow and Cai, 2001; Sparrow et al., 2006; Sparrow et al., 2000; Sparrow et al., 1999; Sparrow et al., 2003a; Sparrow et al., 2002; Sparrow et al., 2003b; Thao et al., 2013; van der Burght et al., 2013; Vives-Bauza et al., 2008; Wang et al., 2006; Washington et al., 2005; Wielgus et al., 2010; Wu et al., 2010; Yoon et al., 2012; Yoon et al., 2011; Zhou et al., 2006), few data demonstrate that these dimers are able to induce morphological and functional changes observed during the onset and progression of AMD.

Although the underlying mechanisms responsible for age-related macular degeneration are unclear, more is known about its morphological progression (Del Priore et al., 2002; Gao and Hollyfield, 1992; Harman et al., 1997; Kliffen et al., 1997; Panda-Jonas et al., 1996; Sarks et al., 1988; Young, 1987). AMD-related retinal degeneration starts with an early accumulation of deposits in and between the RPE and BM. End stages are marked by atrophy of outer and inner segments followed by the remaining retinal layers (Curcio et al., 1993; Jackson et al., 1999; Owsley et al., 2001). The growth of blood vessels from the choroid into the retina can also be a complication (exudative AMD).

To determine whether the age-related increase in vitamin A dimers could trigger morphological changes associated with AMD, we devised a chromatography-free method to prepare gram quantities of the A2E vitamin A dimer and investigated the consequences of introducing it into the eye of the rabbit via intravenous injection in the marginal ear vein. Exogenous administration of A2E resulted in retinal degeneration, as measured by electroretinography and confirmed by light microscopy. The data reveal that A2E and other vitamin A dimers formed in the human eye can potentially be the primary life-long assaults that lead to retinal death, and suggest that methods to prevent the formation of these dimers might address an underlying cause of retinal degeneration and prevent some of the most prevalent causes of blindness.

**TRANSLATIONAL IMPACT**

**Clinical issue**

The eye uses vitamin A as a cofactor to sense light. During this process, a fraction of vitamin A dimerizes, forming vitamin A dimers. A striking chemical signature of retinas undergoing degeneration in major eye diseases such as age-related macular degeneration (AMD) and Stargardt disease is the accumulation of these dimers in the retinal pigment epithelium (RPE) and Bruch’s membrane (BM; an extracellular matrix structure located between the retina and the vascular layer of the choroid). However, it is not known whether the accumulation of dimers of vitamin A is a secondary symptom or a primary driver of degeneration in these diseases. Answering this question has been hampered by the lack of models to study the effects of dimerized vitamin A on retinal health.

**Results**

In this study, the authors present a chromatography-free method to prepare gram quantities of a vitamin A dimer, A2E, and show that intravenous administration of A2E to the rabbit results in loss of retinal function, accumulation of debris in the retina and retinal degeneration. In particular, A2E induces inflammation and remodeling of blood vessels in the RPE and the supporting BM, as well as damage to photoreceptors. These data suggest that vitamin A dimers are not bystanders, but can be primary drivers of retinal degeneration.

**Implications and future directions**

The presented data suggest that preventing vitamin A dimerization could be a preemptive strategy to address serious forms of blindness. Exogenous administration of A2E to the retina constitutes a controlled, rapid injury model of retinal degeneration, which might provide a useful platform to understand the progression of retinal degeneration and to develop therapies.

## RESULTS

### Expedient synthesis of gram-scale A2E

**Fig. 1.**
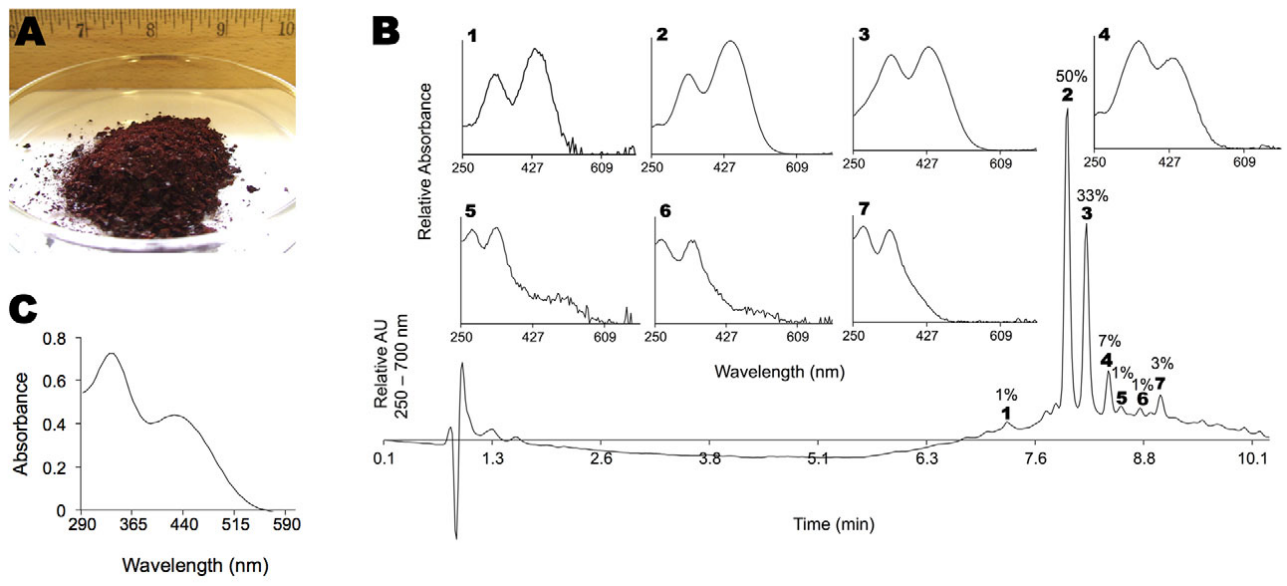
**Expedient synthesis of gram-scale A2E.** (A) Three grams (3 g) of A2E isolated as an amorphous solid by liquid partitioning. Scale provided by ruler graded in inches. (B) Representative HPLC chromatogram of A2E. Labeled peaks (1–7) and corresponding absorption spectra are shown. Percentages are percent areas. Peaks below 2 minutes are from the solvent front. AU, absorption units. (C) Representative UV-Vis spectrum of A2E purified by liquid partitioning.

Fig. 1A depicts 3 g of A2E prepared using liquid partitioning. A2E was isolated as a dark-red solid consisting of a mixture of isomers of A2E. Fig. 1B shows a representative chromatogram from high-performance liquid chromatography (HPLC), with UV detection between 250 and 700 nm, along with the absorbance spectra for select peaks. All-*trans*-A2E and *iso*-A2E are labeled 2 and 3, respectively. Several peaks with comparable retention times to all-*trans*- and *iso*-A2E (i.e. 1, 4–7) exhibited similar and characteristic double-arm absorbance spectra. These peaks represent other geometric isomers of A2E: up to 14 such isomers, in addition to *iso*-A2E, are theoretically possible (Ben-Shabat et al., 2002b). The shorter polyene arm can exist in the all-*trans*, 9- or 11-*cis* configuration, and the longer arm can exist as the all-*trans*-, 9-, 11-, 13-*cis* and 9,13-di-*cis* configurations. Because such peaks are present in chromatograms of extracts from aged human eyes and from mice with the genetic mutation responsible for human Stargardt disease, *ABCA4* knockout animals (supplementary material Fig. S1), we did not attempt further purification. Fig. 1C shows a representative UV-VIS trace of A2E purified by liquid partitioning. Two peaks, at 330 and 425 nm, are observed. The spectrum represents the average absorption spectra of all peaks shown in the HPLC chromatogram in panel B. Using a molecular weight of 652 for the acetate salt, we measured molar extinction coefficients of: ε_330nm_=25,094 M^−1^cm^−1^ and ε_425nm_=15,146 M^−1^cm^−1^ in ethanol.

### Uptake of intravenous A2E by the rabbit RPE

**Fig. 2.**
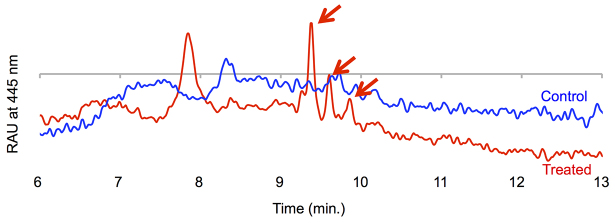
**Intravenously injected A2E reaches the RPE layer.** Representative HPLC traces of organic extracts from an 11-mm-diameter section of the RPE and choroid, from a 7-week-old New Zealand rabbit administered 10 mg of A2E per kg of body weight intravenously 7 days prior to sacrifice (red curve), and from a non-injected 7-week-old control rabbit (blue curve). A2E is seen as a series of peaks highlighted by the arrows. RAU, relative absorption units. A2E retention times differ compared with Fig. 1 because different HPLC conditions were used (see Materials and Methods).

We were able to detect A2E in organic extracts of the RPE and choroid of a 6-week-old rabbit injected intravenously with 10 mg of A2E per kg of body weight 7 days prior (Fig. 2). No A2E was found in age-matched, non-injected rabbits, suggesting uptake of the injected material by the RPE. We thus treated five 6-week-old rabbits with weekly injections of 10 mg of A2E per kg of body weight for 12 weeks and evaluated physiological and morphological changes in the retina. At 7 days after the last injection of A2E, we were able to detect threefold more A2E, as determined by HPLC peak area, in the RPE/choroid compared with age-matched (18-week-old) non-injected control rabbits.

### Retinal function by electroretinography

**Fig. 3.**
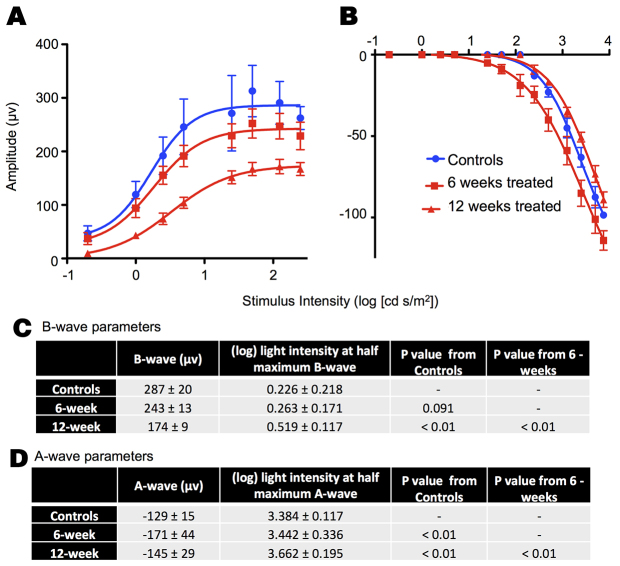
**A2E induces physiological retinal damage.** Average b-wave (A) and a-wave (B) amplitudes in response to flashes of light of increasing intensity for rabbits treated with A2E for 6 weeks (*n*=5) or 12 weeks (*n*=5), along with control rabbits (*n*=3). (C,D) Tables summarizing averages and standard deviations for select ERG parameters.

Fig. 3 shows average electroretinogram (ERG) responses elicited by light of increasing intensity for rabbits before administration of A2E and after A2E was administered intravenously, weekly, over 6 and 12 weeks. The ERG measures the electrical response of the eye elicited by flashes of light. The parameters of the ERG potential, mainly the height of the a- and b-waves, are used clinically and in animal models to evaluate retinal health. Untreated 6-week-old rabbits had a maximum b-wave of ~287 μV. After 6 weeks of treatment, the maximum b-wave was decreased by 15% (Fig. 3A). After an additional 6 weeks of treatment, the maximum b-wave was 40% lower than that of controls. The intensity of light needed to elicit half the maximum b-wave was 16% and 130% greater in 6-and 12-week A2E-treated animals, respectively, compared with the non-treated animals. Changes in the a-wave, thought to reflect the initiation of the visual signal in the photoreceptors, were less pronounced and did not follow a clear pattern (Fig. 3B).

### Morphological changes induced by A2E

**Fig. 4.**
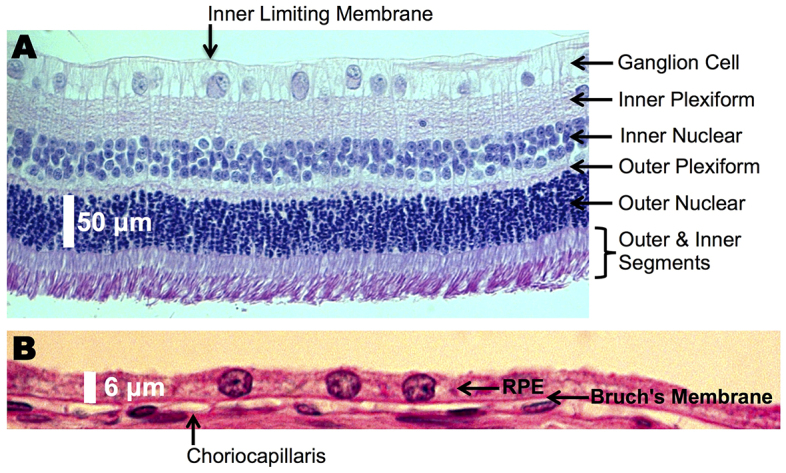
**A normal rat retina.** Representative cross-section of the neural retina (A) and retinal pigment epithelium (RPE; B) from an untreated rabbit, showing normal morphology. Retinal layers are listed. Periodic acid–Schiff stain was used.

To evaluate morphological changes induced by A2E, we examined four of the treated eyes using light microscopy. We employed four common staining methods: (1) toluidine blue, (2) periodic acid–Schiff stain, (3) hematoxylin and eosin (H&E) and (4) macrophage-specific staining. For controls, we evaluated four eyes from untreated age-matched rabbits. The gross structure of the rabbit retina is essentially the same as that of humans. However, the rabbit retina does not have a macula but a visual streak, a horizontally extended area centralis with a higher cell density. Fig. 4 shows a representative section for a control rabbit. In the rabbit, retinal thickness – measured from the inner limiting membrane to the end of the outer and inner segments – varies with location. The retina was thickest in the visual streak (120–160 μm) and became thinner under the medullated fibers and near the serrated junction between the retina and the ciliary body (35–90 μm). The average height of the RPE was about 6 μm, in accordance with literature reports (Ts’o and Friedman, 1967). In addition, retinal vessels in the rabbit extend temporally and nasally, as opposed to in humans, in which the vessels extend over the entire fundus.

**Fig. 5.**
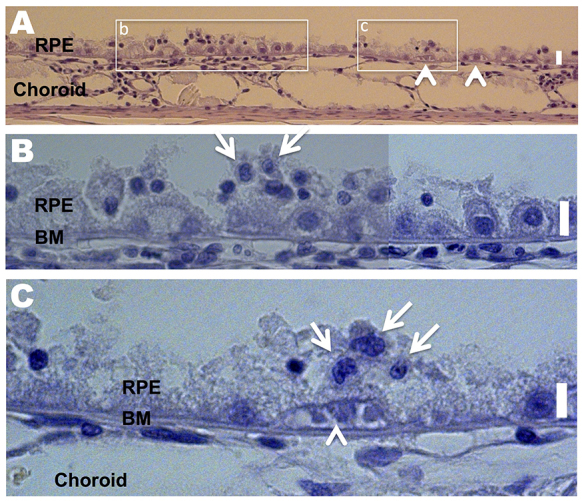
**A2E induces morphological damage to the RPE and choroid.** Toluidine blue staining was used. All scale bars are 6 μm. (A-C) Retina section depicting the RPE and choroid of an A2E-treated rabbit. (B,C) Expanded view of the boxed areas in A. Arrows in B and C point to inflammatory cells with large nuclei and little cytoplasmic space. Note that B is a composite of two images. The arrowheads in A and C point to a capillary-like structure between the RPE and Bruch’s membrane (BM). RPE cells are rounded.

**Fig. 6.**
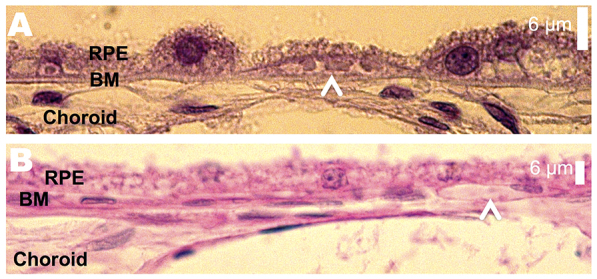
**A2E induces morphological damage to the RPE and choroid.** (A) Atrophied RPE layer with A2E treatment. The arrowhead points to a capillary-like structure between the BM and former RPE layer. Toluidine-blue-stained. (B) A capillary-like structure in the BM (arrowhead). Periodic acid–Schiff-stained.

**Fig. 7.**
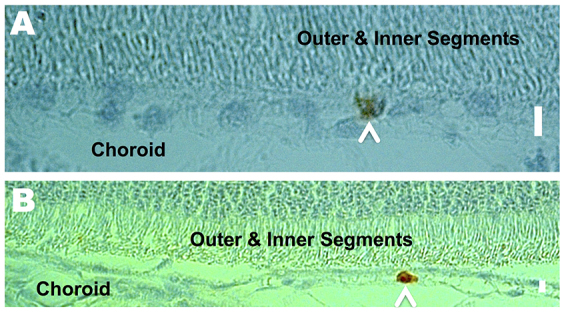
**A2E recruits macrophages.** Representative retinal cross-sections depicting macrophages (arrowheads) in the RPE (A) and choroid (B) of A2E-treated rabbits. Mac-387-stained. Scale bars: 6 μm.

**Fig. 8.**
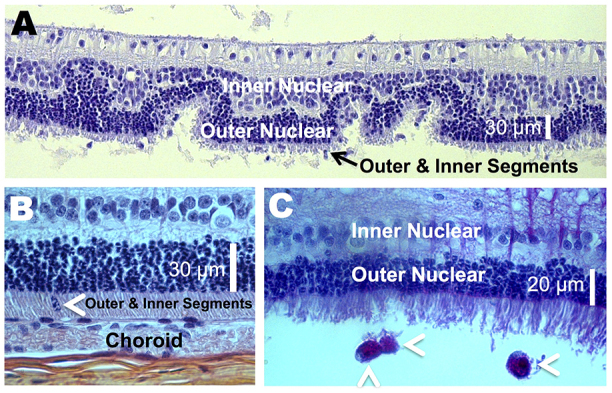
**A2E induces photoreceptor damage.** (A) A disorganized outer nuclear layer, and shortened outer and inner segments, were seen with A2E treatment. Toluidine-blue-stained. (B) A foreign cell infiltrating into the outer and inner segments (arrowhead) is shown. H&E-stained. (C) Three macrophage-like cells (arrowheads) and disrupted outer and inner segments are. Periodic acid–Schiff-stained.

Gross morphological changes induced by A2E were most prevalent in the RPE, choroid, and outer and inner segments. Figs 5–8 depict representative sections illustrating A2E-induced morphological changes. In two of the four eyes imaged, the cell layers of the neural retina were of the normal natural variation for the rabbit. However, for all animals, foreign cells were found in the inner and outer segments (Fig. 8A,B), and portions of the outer and inner segments were disorganized (Fig. 8C), indicating early signs of degeneration. In one of the three eyes imaged, the outer and inner segments were atrophied (Fig. 8A; supplementary material Fig. S2). In general, morphological observations were consistent with changes in the RPE and choroid followed by atrophy of the outer and inner segments, similar to the progression of human AMD (Kliffen et al., 1997; Sarks et al., 1988; Young, 1987).

### Inflammatory cells

Inflammatory cells were present in all of the retinas of treated animals (Figs 5, 7; Fig. 8C). The cells were characterized by their large nuclei and little cytoplasmic space. They could also be identified by periodic acid–Schiff staining, which stains polysaccharides in the cytoplasm of macrophages red to pink (Fig. 8C). Inflammatory cells were located in the RPE, BM, choroid, and outer and inner segments, and were more abundant in areas of increasing damage. We confirmed the presence of macrophages by using a Mac 387 antibody for detection of the myelomonocytic L1 antigen (Brandtzaeg et al., 1988). Macrophages are a key pathological signature of AMD (Jager, 2014; Killingsworth et al., 1990; Seregard et al., 1994). In the particular retina stained, one to two Mac-387-positive cells were detected in the choriocapillaris and/or RPE layer of every slice (Fig. 7, five slices were made and stained). Conversely, no Mac-387-positive cells or inflammatory cells were found in control eyes.

### Subretinal neovascularization

Subretinal neovascularization, where newly formed vessels originate from the choroid and enter the subretinal space, is a common pathological feature of AMD. We were able to observe multiple small vessel-like structures underneath the RPE and/or above the choriocapillaris in all eyes imaged (Fig. 5C; Fig. 6A,B).

### Systemic administration

**Fig. 9.**
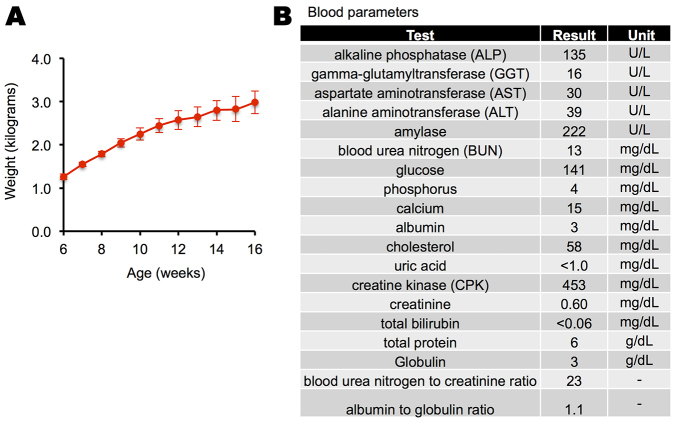
**A2E-treated rabbits seemed otherwise healthy.** (A) Growth curve of five rabbits administered A2E. Averages and standard deviations are shown. (B) Average blood panel for two treated rabbits were in the normal range.

Animals administered A2E showed normal growth (Fig. 9A). For the two animals tested, a comprehensive blood panel remained within normal ranges for the adult rabbit (Fig. 9B) (Hewitt et al., 1989). Treated animals did not show signs of illness or distress (hunching, poor or absent grooming): coats were clean, dry and shiny; animals were well-groomed; the consistency, amount and color of urine and feces were normal; the treated animals displayed normal activity levels; and the animals were bright, alert and reactive.

## DISCUSSION

We developed a chromatography-free method to prepare gram quantities of A2E. The preparation employs a one-step biomimetic preparation from all-*trans*-retinal and ethanolamine, first described by Parish et al. (Parish et al., 1998), but exploits liquid partitioning to isolate A2E. Although the biomimetic preparation has been described, the reaction gives poor yields. Purification of A2E via the biomimetic route involves extensive flash-column and high-pressure chromatography, and, in practice, only a few milligrams of A2E can readily be isolated. There have been attempts to improve this preparation. In 1997, Ren, Sakai and Nakanishi synthesized A2E through a convergent pathway in nine steps (Ren et al., 1997). In 1999, Tanaka and Katsumura reduced the number of steps slightly by incorporating an aza-6π-electrocyclization (Tanaka and Katsumura, 2000), and, in 2005, Sicre and Cid used palladium chemistry to further simplify the preparation of A2E (Sicre and Cid, 2005). However, the aforementioned synthetic routes are still relatively lengthy and large quantities of A2E remained difficult to prepare. We have found that the pyridinium ring of A2E imparts a unique solubility that can be exploited for the isolation of A2E from complex mixtures. By partitioning the crude reaction mixture between three immiscible solvents – 1 M aqueous sodium acetate, acetonitrile and hexane – we were able to separate A2E from its unwanted side products, giving a chromatography-free route to prepare gram quantities of A2E in 50% overall isolated yield.

In the eye, vitamin A dimers form non-enzymatically on primary amines. Dimerization on the amine of phosphatidylethanolamine and subsequent cleavage of the phosphate bond yields A2E. This dimer accumulates with age in the RPE and BM and can be found in the neural retina. Although other dimers exist, A2E has been used as a biomarker of vitamin A dimerization and retinal health because it is relatively easy to quantify. Accumulation of A2E might result from an imbalance of synthesis and/or clearance rates, which may or may not vary with age, retina health and location within the retina. Here, we investigated whether exogenously delivered A2E could trigger retinal degeneration. Although the route of exposure to A2E investigated here differs from how the human retina is exposed, in humans retinal degeneration can take decades. Thus, attempting to replicate *in vivo* fluxes of A2E or its delivery routes is perhaps futile, besides being largely unknown. Here, we investigated intravenous delivery, because this route of delivery does not directly damage the retina or structures of the eye.

The morphological electrophysiological changes observed here, using dimerized vitamin A as a toxicant, are similar to changes reported in response to iodoacetic acid (Campochiaro and Blaydes, 1988; Lachapelle et al., 1990) and sodium iodate (Ashburn et al., 1980; Noell, 1954; Sorsby, 1941). Notably, intravenous administration of these toxicants results in damage to the RPE, choroid and photoreceptors and early b-wave degeneration, while sparing or transiently increasing the a-wave. Significant reductions in the maximum dark-adapted b-wave and small to insignificant changes in the maximum a-wave have also been reported in AMD (Walter et al., 1999), although full-field electroretinograms can vary in AMD depending on the stage of the disease (Jackson et al., 2004; Jackson et al., 2006). Such toxicants have been valuable in understanding retinal physiology and degeneration, and in evaluating interventions. Here, however, vitamin A dimers are naturally present in the human eye and increase with age. Thus, the demonstration that they can directly lead to retinal degeneration in a controlled rabbit model suggests that their accumulation could also trigger similar degeneration in humans. Like the other frequently used toxicants, the administration of dimerized vitamin A thus provides a model to better understand what environments might drive the degenerative process.

A2E induced structural changes and triggered morphological signatures of inflammation and remodeling consistent with angiogenesis in the RPE and the supporting BM, before damaging the neural retina. Chronic inflammation is thought to be a major contributory factor to the development of AMD, and subretinal neovascularization is a contributory factor to vision loss in late wet-AMD. The initial triggers for both processes are not known. Our data suggest that dimerized vitamin A can lead directly to both environments. The *in vivo* morphological response to A2E presented here is in agreement with literature reports in cell models suggesting that A2E can trigger inflammatory cytokines (Anderson et al., 2013; Ma et al., 2013; Sparrow, 2010; Zhou et al., 2006) and vascular growth factors (Anderson et al., 2013; Iriyama et al., 2008; Iriyama et al., 2009).

A2E also induced a gradual retinal degeneration over 3 months. This slow time course is consistent with human retinal degeneration, which typically happens over the course of decades, with gradually progressive pathology (Kliffen et al., 1997; Sarks et al., 1988; Young, 1987). A2E is considered a ‘by-product’ of the visual cycle and is thought to accumulate with age (Eldred and Katz, 1988; Eldred and Lasky, 1993; Sakai et al., 1996). It is tempting to propose that the slow time course of retinal degeneration reflects the slow rate of A2E accumulation, coupled with the slow time course by which it induces cell death.

If A2E is a primary driver of retinal degeneration, a fundamental question is whether those who develop AMD simply have more vitamin A dimers or an aberrant response to vitamin A dimers; data suggest the latter (Radu et al., 2014). Several groups have demonstrated that A2E can induce toxicity and cell death by various mechanisms (Anderson et al., 2013; Avalle et al., 2004; Ben-Shabat et al., 2002a; Ben-Shabat et al., 2002b; Bergmann et al., 2001; De and Sakmar, 2002; Dontsov et al., 2009; Finnemann et al., 2002; Holz et al., 1999; Iriyama et al., 2008; Iriyama et al., 2009; Kanofsky et al., 2003; Lakkaraju et al., 2007; Lukiw et al., 2006; Ma et al., 2013; Moiseyev et al., 2010; Murdaugh et al., 2009; Murdaugh et al., 2010; Nowak, 2013; Radu et al., 2004; Roberts et al., 2002; Sokolov et al., 2007; Sparrow, 2010; Sparrow and Cai, 2001; Sparrow et al., 2006; Sparrow et al., 2000; Sparrow et al., 1999; Sparrow et al., 2003a; Sparrow et al., 2002; Sparrow et al., 2003b; Thao et al., 2013; van der Burght et al., 2013; Vives-Bauza et al., 2008; Wang et al., 2006; Washington et al., 2005; Wielgus et al., 2010; Wu et al., 2010; Yoon et al., 2012; Yoon et al., 2011; Zhou et al., 2006). The exact mechanisms of vitamin-A-dimer-induced retinal death in an individual might differ based on genetics and environment, in line with the multiple genetic susceptibility factors and environmental components that are thought to determine one’s susceptibility to developing AMD (Swaroop et al., 2009).

Several approaches are being considered or are undergoing clinical testing to prevent the dimerization of vitamin A or clear the deposited dimers (Julien and Schraermeyer, 2012; Nociari et al., 2014) as a means of preventing vision loss. Elucidating the mechanisms by which A2E causes toxicity is potentially of diagnostic, predictive and therapeutic value. Delivering A2E to the eye, either intravenously (as done here), intravitreally or in time-release formulations, will serve as a tool to study the consequences of lifelong accumulation of vitamin A dimers in the human retina. The straightforward preparation of A2E presented here will allow further studies mapping signatures of the degenerating retina, such as angiogenesis (Ng and Adamis, 2005), inflammation (Rodrigues, 2007), mitochondriopathy (Karunadharma et al., 2010) and dysregulated autophagy (Kaarniranta et al., 2013), to A2E concentrations, and morphological and functional changes. Although no animal model exists that replicates all the clinical, morphological and chemical signatures of the human condition, neither does any individual. Furthermore, the multifactorial nature of AMD makes the construction of genetically engineered animal models that replicate AMD difficult. However, injury models of retinal disease, such as the laser-induced choroidal neovascularization (Ryan, 1979), have proven valuable in the development of vision-saving therapies. As such, data presented here provide an early rationale for a vitamin-A-dimer-induced injury model of age-related retinal degeneration. Our current findings using this model support the development of methods to retard the dimerization of vitamin A as a viable strategy for a disease-modifying therapeutic to prevent blindness.

## MATERIALS AND METHODS

### Preparation of A2E

All manipulations were done under safelights (lighting above >550 nm). We added, to a flask, 8 g of retinaldehyde, 250 ml anhydrous ethanol, 0.5 mol equivalents of ethanolamine and 0.6 mol equivalents of glacial acetic acid. The reaction was stirred for 3 days in the dark. To isolate A2E, we evaporated the ethanol from the reaction mixture by rotary evaporation and re-solvated the resulting gum in acetonitrile. Less-polar constituents, such as all-*trans*-retinaldehyde, the retinaldehyde dimer, retinaldehyde Schiff base and various unknown constituents, were removed by washing the acetonitrile with equal volumes of hexane in a separatory funnel (four times). More-polar constituents, such as ethanolamine and acetic acid, were removed by washing the acetonitrile with equal volumes of both an aqueous 1 M sodium acetate solution and hexane (four times), collecting the middle acetonitrile layer containing A2E each time. Each layer from each round of washing was monitored by HPLC. After washing, the acetonitrile was evaporated and trace solvent was removed by overnight high vacuum.

Chromatography was performed using a Waters (Waters, Inc., Milford, MA) HPLC system featuring a 996 photodiode array detector, Model 600 pump and Model 600 controller equipped with an in-line degasser. Data were processed using Empower Pro (v 5.0) software (Waters). Reversed-phase HPLC was performed at room temperature using an Onyx Monolithic C18 column (100 cm, 3 mm I.D.) (Phenomenex^®^ Inc., Torrance, CA). The mobile phase was flowed at 2 ml per minute and started with 75% acetonitrile containing 0.05% trifluoroacetic acid (Pump A) and 25% deionized water also containing 0.05% trifluoroacetic acid (Pump A) and was increased linearly to 100% acetonitrile containing 0.05% trifluoroacetic acid over 10 minutes and then eluted for an additional 10 minutes. A2E liposomes were prepared as described (Mihai and Washington, 2014), scaled accordingly using 1 g A2E. The final concentration of A2E was 10 mg/5 ml solution.

### Administration of A2E

Columbia’s Institutional Animal Care and Use Committee approved animal protocols. We used five New Zealand albino rabbits (Harlan Laboratories, Indianapolis, IN), 6 weeks of age, weighing an average of 1.3 kg. The rabbits were housed under a 12/12-hour light/dark cycle with free access to food and water. We weighed animals weekly and observed them and their cages daily. A2E (10 mg/kg body weight) in a liposomal solution (Mihai and Washington, 2014) was administered weekly through the marginal ear vein.

### A2E quantification

Animals were euthanized with Ethasol, eyes enucleated and dissected to give the eyecup. An 11-mm diameter full-eye wall punch (through the retina, choroid and the supporting sclera) was taken. The punch was homogenized, extracted with ethanol (300 μl) and analyzed by HPLC as previously described (Ma et al., 2011).

### ERG

ERG recordings were performed as previously described (Ma et al., 2011; Washington et al., 2007) but using flashes provided by a xenon lamp, ranging from 0.2 to 7500 cd s/m^2^, scotopic units, in 13 steps. For each animal, ERG potentials were recorded from both eyes. Traces that were judged excessively noisy were rejected. At least two ERG curves were averaged for each step and eye. Traces from both eyes of each animal were then averaged to give an ERG response for each probe flash for each step per animal. Using GraphPad Prism (La Jolla, CA), we then fit a- and b-wave responses for each light intensity and each animal to a sigmoidal curve (Low, 1987) described by: *Y*=Bottom + (Top–Bottom)/(1+10^[(LogEC50–*X*)×HillSlope)], where: Top is the maximum a- or b-wave, in the same units as *Y*; LogEC50 is the intensity needed to achieve a half-maximum a- or b-wave, in the same units as X; and HillSlope is a parameter that describes the steepness of the dose-response curve. *P*-values were calculated with an F-test.

### Histology

Whole eyes were fixed for 1 week in Carnoy’s fluid fixative, embedded in paraffin, sectioned vertically through the optic nerve (superior-inferior) and stained with either: (1) toluidine blue, (2) periodic acid–Schiff stain, (3) H&E or (4) an anti-macrophage antibody (MAC387, Thermo Scientific, Fremont, CA).

## Supplementary Material

Supplementary Material
