# Supplementary material for: Morphological and physiological retinal degeneration induced by intravenous delivery of vitamin A dimers in rabbits
Source: Dis Model Mech. 2014 Dec 12;8(2):131–8. doi: 10.1242/dmm.017194 (PMC4314778; doi:10.1242/dmm.017194)
Supplement: Supplementary Material [file supp_8_2_131__index.html]

Morphological and physiological retinal degeneration induced by intravenous delivery of vitamin A dimers in rabbits — Supplementary Material 

# Morphological and physiological retinal degeneration induced by intravenous delivery of vitamin A dimers in rabbits

## DMM017194 Supplementary Material

**Files in this Data Supplement:**

- **Supplementary Material**
