# Supplementary material for: Morphological and physiological retinal degeneration induced by intravenous delivery of vitamin A dimers in rabbits
Source: Dis Model Mech. 2014 Dec 12;8(2):131–8. doi: 10.1242/dmm.017194 (PMC4314778; doi:10.1242/dmm.017194)
Supplement: Supplementary Material [file supp_8.2.131_DMM017194.pdf]

Morphologic and physiologic retinal degeneration induced by intravenous delivery of vitamin A dimers in the leporid retina

Jacky Penn, Doina M. Mihai and Ilyas Washington*

Columbia University Medical Center, Ophthalmology, New York, NY 10032, USA.

Supplementary Material

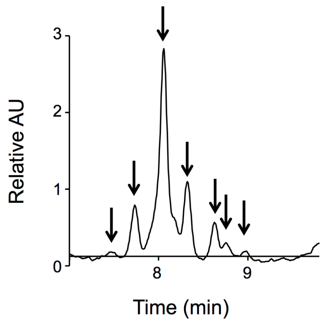

Fig. 1 Representative HPLC chromatogram of an ethanolic extract of eyecups from eight, 6-month old, ABCA4$^{-/-}$ mice. The spectrum is the average of all spectra when scanned from 250-700 nm. Several peaks are shown (arrows) representing dimerized vitamin A. The distribution of peaks is similar to what is obtained when A2E is prepared and isolated by liquid partitioning. AU: absorption units

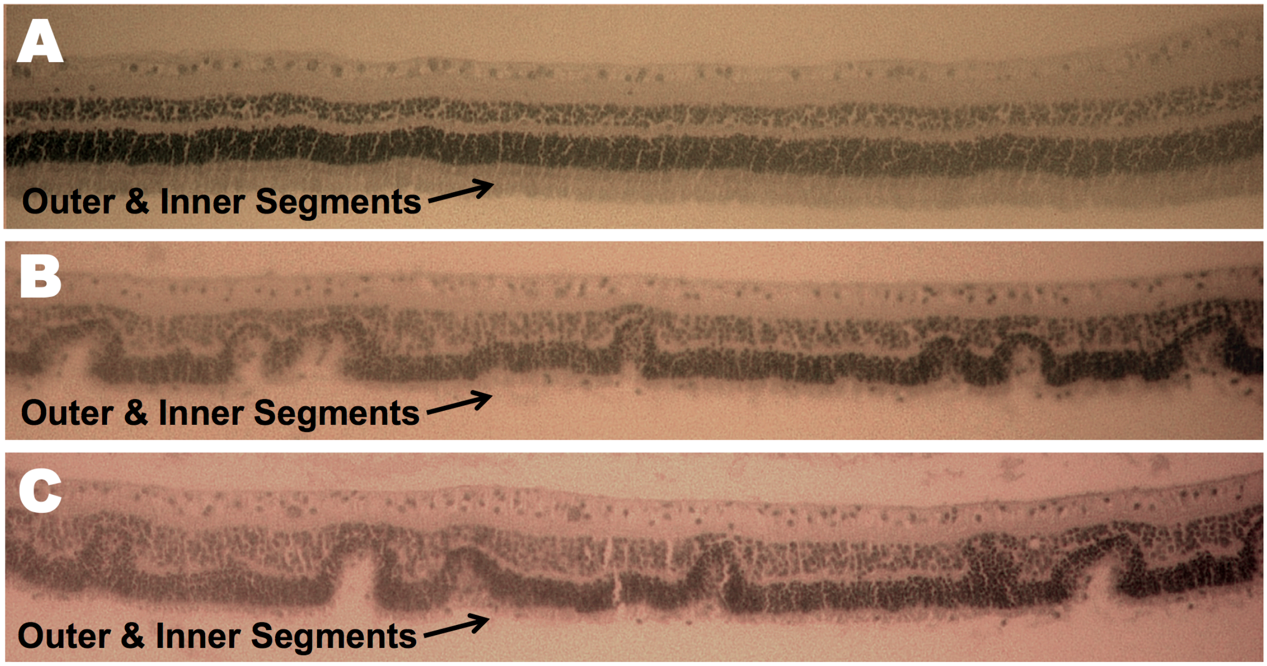

Fig. 2 Representative retinal cross sections from a control (**A**) and A2E treated (**B** and **C**) rabbit taken at 10X magnification showing shortened outer and inner segments and rosette-like structures. Toluidine blue stained.
